# Exosome-mediated delivery of super-repressor IκBα alleviates inflammation and joint damages in rheumatoid arthritis

**DOI:** 10.1186/s13075-023-03225-1

**Published:** 2024-01-02

**Authors:** Hae-In Lee, Min-Joo Ahn, Jae-Kwang Yoo, So-Hee Ahn, Seon Young Park, Hyangmi Seo, Moon-Ju Kim, Yu Jeong Lee, Hyun Hee Jang, Seung Cheol Shim, Eun Jeong Won, Cheolhyoung Park, Chulhee Choi, Tae-Jong Kim

**Affiliations:** 1 Department of Rheumatology, Chonnam National University Medical School and Hospital, Gwangju, 501-757 Republic of Korea; 2 Department of Biomedical Sciences, Graduate School of Chonnam National University, Gwangju, Republic of Korea; 3 Division of Rheumatology, Daejeon Rheumatoid & Degenerative Arthritis Center, Chungnam National University Hospital, Daejeon, Republic of Korea; 4 ILIAS Biologics Inc, Daejeon, Republic of Korea; 5 Department of Laboratory Medicine, Asan Medical Center, University of Ulsan College of Medicine, Seoul, Republic of Korea

**Keywords:** Rheumatoid arthritis, Exosome, Inflammation, NF-κB, Treatment

## Abstract

**Background:**

This study aims to investigate the potential anti-inflammatory effects of exosomes engineered to carry super-repressor IκB (Exo-srIκB), an exosome-based NF-κB inhibitor, in the context of RA.

**Methods:**

Peripheral blood mononuclear cells (PBMCs) and synovial fluid mononuclear cells (SFMCs) were collected from patients diagnosed with RA and treated with Exo-srIκB to test the therapeutic potential. Flow cytometry analysis was performed to assess the production of inflammatory cytokines (IL-17A and GM-CSF) by the cells. ELISA was utilized to measure the levels of TNF-α, IL-17A, IL-6, and GM-CSF. Arthritis was induced in SKG mice by intraperitoneal injection of curdlan. DBA/1 J mice were used in collagen-induced arthritis (CIA) experiments. After the development of arthritis, mice were injected with either Exo-Naïve (control exosome) or Exo-srIκB. Arthritis scores were recorded biweekly, and histological observations of the ankle joint were conducted using H&E and safranin-O staining. Additionally, bone erosion was evaluated using micro-CT imaging.

**Results:**

In the ex vivo study involving human PBMCs and SFMCs, treatment with Exo-srIκB demonstrated a notable reduction in inflammatory cytokines. Furthermore, in both the SKG and CIA models, Exo-srIκB treatment exhibited significant reductions in inflammation, cartilage destruction, and bone erosion within the joint tissues when compared to the Exo-Naive control group. Additionally, the radiographic score assessed through microCT showed a significant decrease compared to the Exo-Naive control group.

**Conclusion:**

Overall, these findings suggest that Exo-srIκB possesses anti-inflammatory properties in human RA cells and animal models, making it a promising therapeutic candidate for the treatment of RA.

**Supplementary Information:**

The online version contains supplementary material available at 10.1186/s13075-023-03225-1.

## Background

Rheumatoid arthritis (RA) is a chronic inflammatory disorder characterized by synovial hyperplasia, angiogenesis, inflammatory cell infiltration, pannus formation, cartilage destruction, and bone erosion. As RA progresses, it can lead to joint destruction and disability [1].

The nuclear factor-κB (NF-κB) is a family of inducible transcription factors that regulates numerous genes involved in immune and inflammatory responses. Various factors, such as IL-1, IL-17, TNF-α, platelet-derived growth factor, lipopolysaccharide, oxidative stress, and viral products, can induce NF-κB activation. NF-κB, in turn, triggers the transcription of IL-1, TNF-α, IL-6, IL-8, IL-17, GM-CSF, and inducible nitric oxide synthase [2]. NF-κB plays a significant role in RA pathology [3], and novel therapeutic strategies aimed at specific inhibition of key elements in the NF-κB activation pathway have been under development in recent years [4, 5].

Exosomes are small membrane-enclosed vesicles released by cells for intercellular communication. They are derived from the fusion of multivesicular bodies with the cell membrane [6]. Exosomes, as biologically derived nanoparticles, offer efficient drug delivery and excellent biocompatibility with minimal side effects. They can elicit robust cellular responses both in vitro and in vivo, making them promising therapeutic agents [7, 8]. NF-kB exists in an inactive state within the cytoplasm of nearly all mammalian cells, and it forms associations with inhibitory proteins collectively known as IκB (inhibitory κB proteins). Our group has previously reported that delivering exosomal super-repressor IκB (Exo-srIκB) using “EXPLOR” (exosomes for protein loading via optically reversible protein–protein interactions) technology [9] can alleviate inflammation in disease models such as sepsis [4] and acute kidney injury [5]. However, the potential of delivering srIκB using exosomes in RA remains unexplored.

With this in mind, we employed the same technology to load srIkB into exosomes and deliver them systemically as Exo-srIkB to assess their impact on RA. This study aimed to investigate the efficacy of Exo-srIκB in alleviating arthritis, bone damage, and inflammation using human ex vivo samples and mouse models of RA.

## Materials and methods

### Human samples

All patients met the RA criteria established by the American College of Rheumatology/European League Against Rheumatism [10]. Peripheral blood mononuclear cells (PBMCs) and synovial fluid mononuclear cells (SFMCs) were collected from active RA patients. The demographic characteristics of the patients are provided in Table 1. The study received approval from the Ethics Committee of Chonnam National University Hospital (CNUH). Written informed consent was obtained from all participants (institutional review board [IRB] no. CNUH-2011–199).

**Table 1:** Clinical characteristics and laboratory findings of patients with RA

	PBMC	SFMC
Total number	9	6
Age, mean ± SD (years)	60.8 ± 19.2	52.5 ± 20.3
Male, *n* (%)	3 (33.3)	1 (16.6)
RF positive, *n* (%)	8 (88.8)	5 (83.3)
Anti-CCP Ab positive, *n* (%)	8 (88.8)	5 (83.3)
Recent medications		
Steroid, *n* (%)	7 (77.7)	6 (100.0)
Steroid dose, mean ± SD	5.4 ± 3.4	6.6 ± 2.7
Methotrexate use, *n* (%)	9 (100.0)	6 (100.0)
Leflunomide use, *n* (%)	3 (33.3)	1 (16.6)
Tacrolimus use, *n* (%)	3 (33.3)	2 (33.3)
TNF-α blocker use, *n* (%)	2 (22.2)	0 (0.0)
Jak inhibitor use, *n* (%)	0 (0.0)	0 (0.0)

### Production of Exo-srIκB

The process of exosome production was previously described [11, 12]. Briefly, Expi293F-producing cells were incubated in a wave culture system for 4 days and exposed to blue light for target protein loading and exosome production. Next, the harvested culture medium was centrifuged at 2000 g for 10 min to remove cells and debris and filtered with a 0.22-µm polyethersulfone filter to remove large particles. The exosome was subsequently purified using ultrafiltration and diafiltration for concentration, buffer exchange, and anionic and multimodal resin chromatography. Finally, a formulation and sterilization filter process was performed.

### Characterization of Exo-srIκB

The morphology and lipid bilayer of extracellular vesicles (EVs) were absorbed on carbon-coated copper, stained with 2% uranyl acetate, and confirmed by transmission electron microscopy. Nanoparticle tracking analysis was used to measure EVs’ particle number and size distribution using NS300, and samples were diluted (1:100–1:10,000) in particle-free PBS to an acceptable concentration. Immunoblotting was performed by lysing cells in RIPA buffer or exosomes, followed by SDS/PAGE gel electrophoresis and transfer onto nitrocellulose membrane. Membranes were blocked with 5% skim milk in Tris-buffered saline containing 0.1% Tween-20 (TBS-T) and probed with primary antibodies against srIκB, CRY2 (customized antibody, AbClon, Seoul, Korea), CD9, CD81 (SBI, Tokyo, Japan), TSG101, Alix, GM130, calnexin (Abcam, Cambridge, UK), lamin B1, GAPDH (Santa Cruz Biotechnology, Dallas, TX, USA), and prohibitin (Novusbio, Centennial, CO, USA) at 4 °C overnight. After incubation with specific secondary antibodies, blots were developed using Clarity and Clarity Max ECL Western blotting substrates and imaged with the ChemiDoc imager.

### Cell viability assay

Cell viability was assessed using the Cell Titer 96 AQueous One Solution Reagent (G3580, Promega, USA). Cells were seeded and treated with varying concentrations of Exo-srlκB for specified durations. Following the manufacturer’s instructions, 20 μl of MTS solution was added to 100 μl of cell culture medium and incubated at 37 °C for 2–4 h. Absorbance was then measured at 490 nm using a Molecular Devices Reader 96-well microplate reader (USA). For flow cytometry analysis, whole cells were surface stained with anti-Fixable Viability Dye-eFluor780 (65–0865-14, Invitrogen, USA).

### Co-culture of Exo-srIκB with human inflammatory cells

PBMCs and SFMCs were isolated and cultured in RPMI1640 media (LM011-01, Welgene, Korea) supplemented with 10% fetal bovine serum (S001-01, Welgene, Korea) and 1% penicillin–streptomycin solutions (LS202-02, Welgene, Korea). Cells were seeded at a density of 5 × 10^5^ cells/well in a 96-well plate. After a 3-h pretreatment with non-engineered cell-derived control exosomes (Exo-Naïve) or Exo-srIκB, the cells were stimulated with phorbol 12-myristate 13-acetate (PMA; P1585, Sigma, USA) at a concentration of 100 ng/ml, ionomycin (I9657, Sigma, USA) at a concentration of 1 μM, and brefeldin A (a Golgi plug protein transport inhibitor; 555,029, BD, USA). The cells were then incubated in CO_2_, 37 °C incubator for 4 h. Following stimulation, cells were stained with anti-Fixable Viability Dye-eFuor780 (65–0865-14, Invitrogen, USA). After washing, cells were fixed and permeabilized using perm/wash buffer and stained with APC-conjugated anti-IL-17A (512,334, BioLegend, USA) and PerCP-Cy5.5-conjugated anti-GM-CSF (502,312, BioLegend, USA) antibodies. FlowJo Software (BD, USA) was used for flow cytometry analysis. Additionally, supernatants from PBMCs and SFMCs were analyzed for human IL-17A, IL-6, and TNF-α levels using ELISA kits (88–7176, 88–7066, 88–7344, Invitrogen, Austria), and human GM-CSF levels were measured using an ELISA kit (K0331120, LABISKOMA, Korea). Optical density (OD) was measured at 450 nm using a SpectraMax® M2 microplate reader (Molecular Devices Corp., USA).

### Experimental arthritis mouse model, intervention and scoring

Experiments were conducted with the approval of the Institutional Animal Care and Use Committee (animal experiment IRB no. CNU IACUC-H-2021–17). SKG mice on a BALB/c background were obtained from CLEA Japan (Tokyo, Japan) and housed in a specific pathogen-free (SPF) facility. The negative control mice were not injected with curdlan to assess the baseline response in the absence of the experimental intervention (*n* = 9). Eight-week-old female mice were treated with curdlan (3 mg/kg) by intraperitoneal injection. After the onset of symptoms following curdlan injection, the mice were randomly stratified into two groups (*n* = 9 per group): one receiving Exo-Naïve treatment and the other receiving Exo-srIκB treatment. Either Exo-Naïve (1 × 10^10^ pn/0.2 ml) or Exo-srIκB (1 × 10^10^ pn/0.2 ml) was repeatedly administered intraperitoneally three times a week until sacrifice.

Collagen-induced arthritis (CIA) model was prepared in 8–9-week-old male DBA/1 J mice and conducted in a SPF facility. The mice underwent primary immunization followed by a 21-day interval before secondary immunization. Random assignment of experimental groups was performed prior to the study (*n* = 5 mice for each group). Repeated treatments of all the test substances were initiated on day 23. The negative control mice were not immunized, serving as a baseline for assessing the response in the absence of the experimental intervention. The Exo-Naïve (1 × 10^10^ pn/0.2 ml) group received only exosome treatment, while the treatment group received 1 × 10^10^ pn/0.2 ml of Exo-SrlκB, which was administered three times a week until sacrifice. Methotrexate (MTX), as a positive control, was intraperitoneally administered at a dosage of 1 mg/kg per administration, given twice a week.

Clinical signs were monitored and scored twice a week by two independent observers using the following scale for affected joints: 0 = asymptomatic, 1 = slight swelling of ankles or toes, 2 = severe ankle swelling, 3 = severe ankle and toe swelling, and 4 = ankle and toe swelling with twisting [13].

### Tissue preparation and histological analysis

After completing the experiment, mice were sacrificed, and ankle samples were collected. Ankle specimens were fixed in 10% formalin for 1 week, decalcified in 10% formic acid at 37 °C for 1 week with shaking, and then embedded in paraffin. Paraffin blocks were sectioned at a thickness of 3.5 µm and deparaffinized using neo-clear (109,843, Merck, USA). Gradually graded ethanol was used for hydration, followed by staining with hematoxylin (105,174, Merck, USA) and eosin (HT110216, Sigma, USA). Additionally, safranin-O staining was performed on the joints to assess cartilage destruction. Two blinded readers independently scored the histological arthritis samples according to a previous report [14].

### Immunofluorescent staining

The section slides were deparaffinized in neo-clear and rehydrated in serial ethanol, followed by antigen retrieval with proteinase K (Abcam, ab64220) at RT for 30 min and blocking with BLOXALL (Vector, SP-6000) for 1 h. To observe co-localization of CD4, IL-17A, and TNF-α, the slides were incubated with primary mouse antibody for TNF-α (1:100, Santa, sc-52746), rabbit antibody for IL-17A (1:50; Abcam, ab79056), and rat antibody for CD4 (1:50; Santa, sc-19641) at RT for 1 h, followed by incubation with 488-conjugated anti-mouse antibody (1:100; Invitrogen, A11001), Cy3-Alexa-conjugated anti-rabbit antibody (1:100; Jackson ImmunoResearch, 111–165-144), and Cy5-conjugated anti-rat antibody (1:100; Jackson ImmunoResearch, 712–175-153) at RT for 1 h. To avoid nonspecific staining, the stained slides were treated with DAPI using the Autofluorescence Quenching Kit (Vector, SP-8500). Immunofluorescent images were collected by a confocal microscope (Leica Microsystem, Germany).

### Micro-computed tomography analysis

The Quantum FX (μCT, Perkin Elmer) was utilized for imaging purposes. The scanning parameters were configured to 90 kV and 180 uA, with a scan duration of 2 min. The field of view (FOV) encompassed 20 mm, and the resolution achieved was 40 µm. Following the immobilization of the representative foot tissue that best reflected the clinical indicators of each group, a μCT scanner was employed for scanning. Radiographic images were acquired using the Quantum FX μCT imaging system (Perkin Elmer, MA, USA) and subsequently subjected to 3D rendering. Radiographic scoring was conducted, involving the independent evaluation of joint destruction by two researchers [15]. The scoring value was determined by averaging the evaluations of the researchers, and the average scoring value of the foot tissue was computed as the score for each group.

### Statistical analysis

Statistical analysis was conducted using Prism 9.0 Software (GraphPad Software, San Diego, CA, USA). Differences between means were evaluated for statistical significance using various tests, including Kruskal–Wallis test with Dunn’s multiple comparisons, *T*-test, Wilcoxon matched-pairs signed-rank test, two-way analysis of variance (ANOVA), and Mann–Whitney test. Significance levels were indicated on the graphs as follows: *P < 0.05, **P < 0.01, ***P < 0.001, and ****P < 0.0001. A *P*-value less than 0.05 was considered statistically significant.

## Results

### Production and characterization of engineered exosomes

**Fig. 1:**
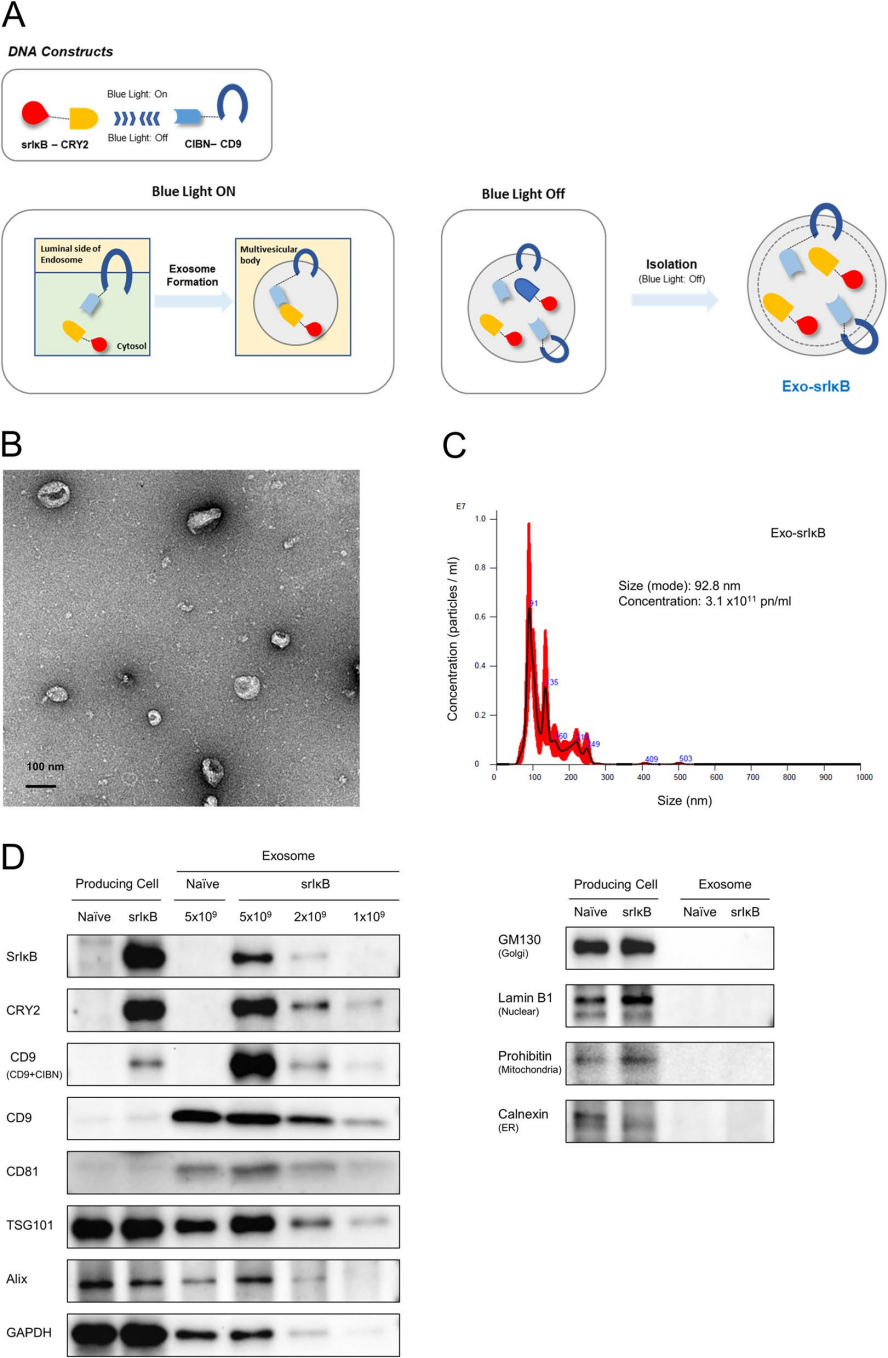
Production and characterization of Exo-srIκB. **A** DNA constructs and blue-light-mediated fusion of recombinant proteins were used to produce Exo-srIκB, as shown in schematic diagrams. **B** Representative images for transmission electron microscopy of Exo-srIκB morphology. **C** Representative panels for concentration and size distribution of Exo-srIκB were determined by a NanoSight (NS300) instrument. **D** Immunoblotting of Expi293F cells (producing cells) and Expi293F cell-derived exosomes to analyze the expression of recombinant proteins (srIκB, CRY2, and CD9) and exosome markers (CD9, CD81, TSG101, Alix, and GAPDH) and cell organelle markers (GM130, lamin B1, prohibitin, and calnexin). Exo-Naïve (non-engineered cell and exosome) were used as negative controls of Exo-srIκB

The top schematic in Fig. 1A depicts the DNA constructs used for producing Exo-srIκB, while the bottom schematic demonstrates fusion proteins and their expected activities through light-dependent protein loading. Exo-srIκB was characterized in terms of morphology using transmission electron microscopy (Fig. 1B). The size and concentration of Exo-srIκB were determined to be 92.8 nm and 3.1 × 10^11^ pn/ml, respectively (Fig. 1C). The expression of recombinant proteins (srIκB, CRY2, and CD9), exosome markers (CD9, CD81, TSG101, Alix, and GAPDH), and cell organelle markers (GM130, lamin B1, prohibitin, and calnexin) was thoroughly characterized (Fig. 1D). We noted that genetic constructs expressing a modified version of the IκB protein, which lacks phosphorylation sites and is termed srIκB, exhibited effective loading. Furthermore, we confirmed that Exo-srIκB suppresses NF-κB phosphorylation (Suppl. Figure 1).

### Assessment of cell viability with Exo-srIκB

**Fig. 2:**
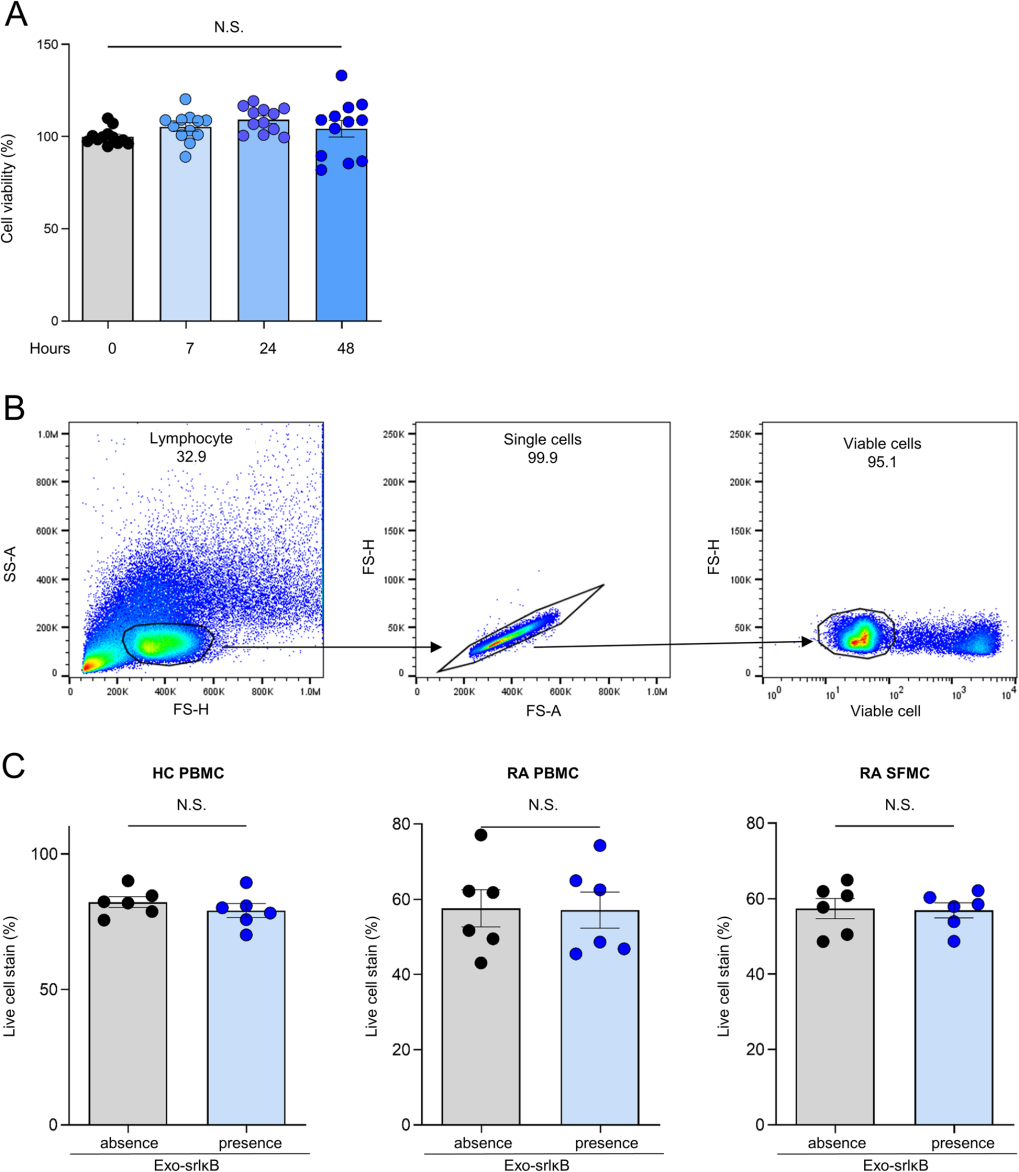
Assessment of cell viability with Exo-srIκB. **A** The cell viability of PBMCs and SFMCs was assessed using the MTS assay, taking into account the duration of Exo-srIκB treatment. Statistical significance was determined using the Kruskal–Wallis test with Dunn’s multiple comparisons. The presented values represent the mean ± SEM. **B** A representative gating strategy for flow cytometry was employed to evaluate the survival rate. **C** Viability dyes were used to stain and measure PBMC and SFMC viability. Statistical significance was determined using a *T*-test. The presented values represent the mean ± SEM, and symbols represent individual sample. NS, not significant

The cell viability was assessed using the MTS assay, taking into account the duration of Exo-srIκB treatment. We observed that administering Exo-srIκB at a concentration of 1 × 10^10^ pn for duration of 7, 24, and 48 h did not significantly impact cell viability (Fig. 2A). Flow cytometry analysis corroborated this, showing no decrease in cell viability for PBMCs and SFMCs as indicated by the gating strategy (Fig. 2B) and subsequent results (Fig. 2C).

### Exo-srIκB exhibits remarkable suppression of inflammatory cytokines

**Fig. 3:**
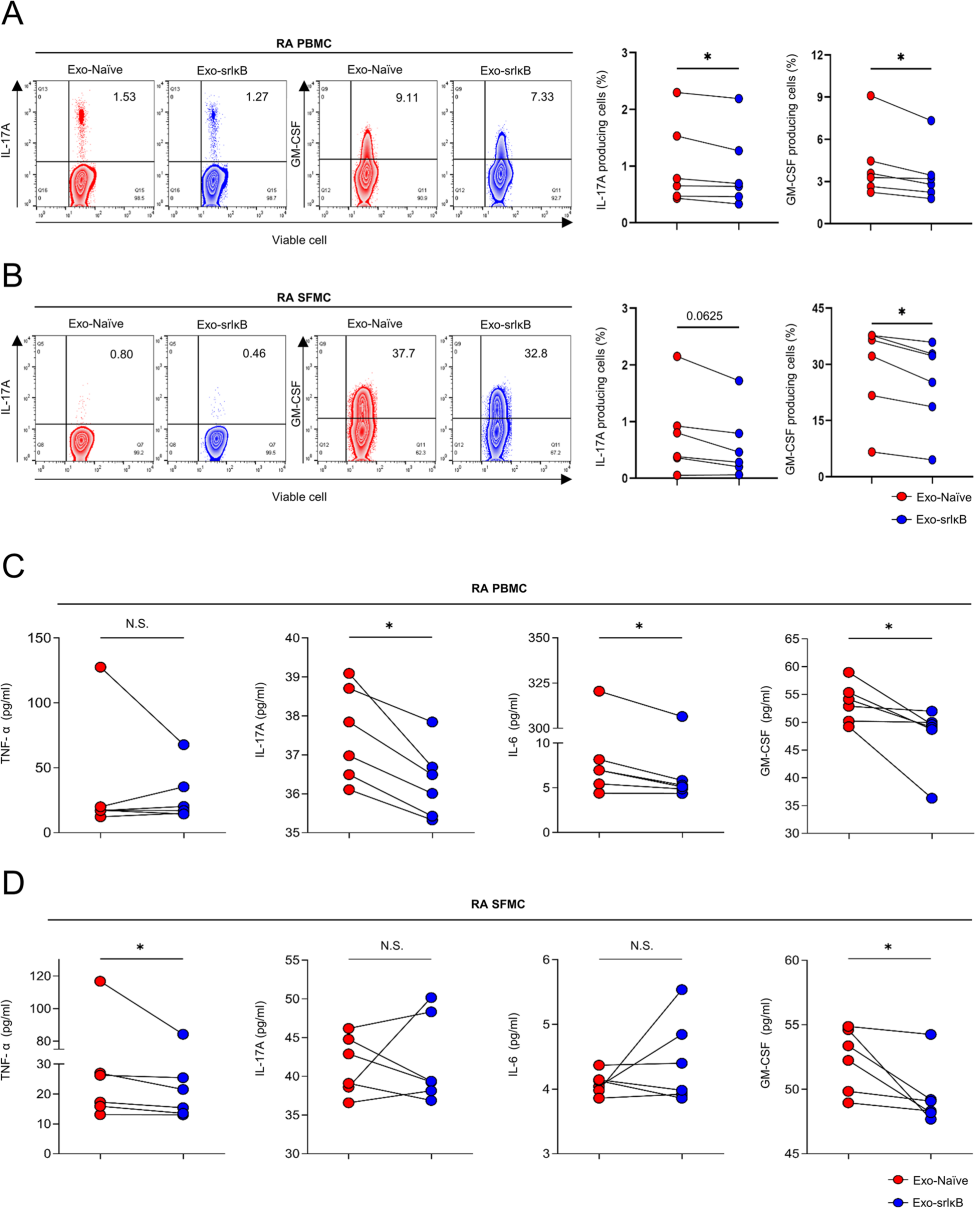
Exo-srIκB suppresses inflammatory cytokines in both PBMCs and SFMCs obtained from patients with rheumatoid arthritis. **A**, **B** Flow cytometry analysis was performed to assess the percentages of IL-17A and GM-CSF-positive cells in PBMCs and SFMCs. **C**, **D** ELISA was conducted to measure TNF-α, IL-17A, IL-6, and GM-CSF levels in ex vivo supernatants from PBMCs and SFMCs. Statistical significance was determined using the Wilcoxon matched-pairs signed-rank test. The symbols represent individual sample. NS, not significant. *P < 0.05

To explore the anti-inflammatory effects of Exo-srIκB, PBMCs and SFMCs from RA patients were stimulated and cultured ex vivo for 7 h in either the Exo-Naïve or Exo-srIκB treatment groups. The gating strategy was demonstrated using flow cytometry analysis (Suppl. Figure 2). The Exo-srIκB treatment significantly reduced the frequencies of IL-17A- and GM-CSF-producing cells in the PBMCs of RA patients compared to the Exo-Naïve treatment (Fig. 3A). In the SFMCs of RA patients, the Exo-srIκB treatment showed a trend of decreasing the frequency of IL-17A-producing cells, and it significantly lowered the frequency of GM-CSF-producing cells compared to the Exo-Naïve treatment (Fig. 3B). The Exo-srIκB treatment also significantly reduced the levels of IL-17A, IL-6, and GM-CSF in the culture medium of RA PBMCs (Fig. 3C). Additionally, in the culture medium of SFMCs, Exo-srIκB treatment significantly diminished the levels of TNF-α and GM-CSF (Fig. 3D).

### Exo-srIκB treatment suppresses clinical arthritis and decreases inflammatory cell infiltration in the joints of SKG mice

**Fig. 4:**
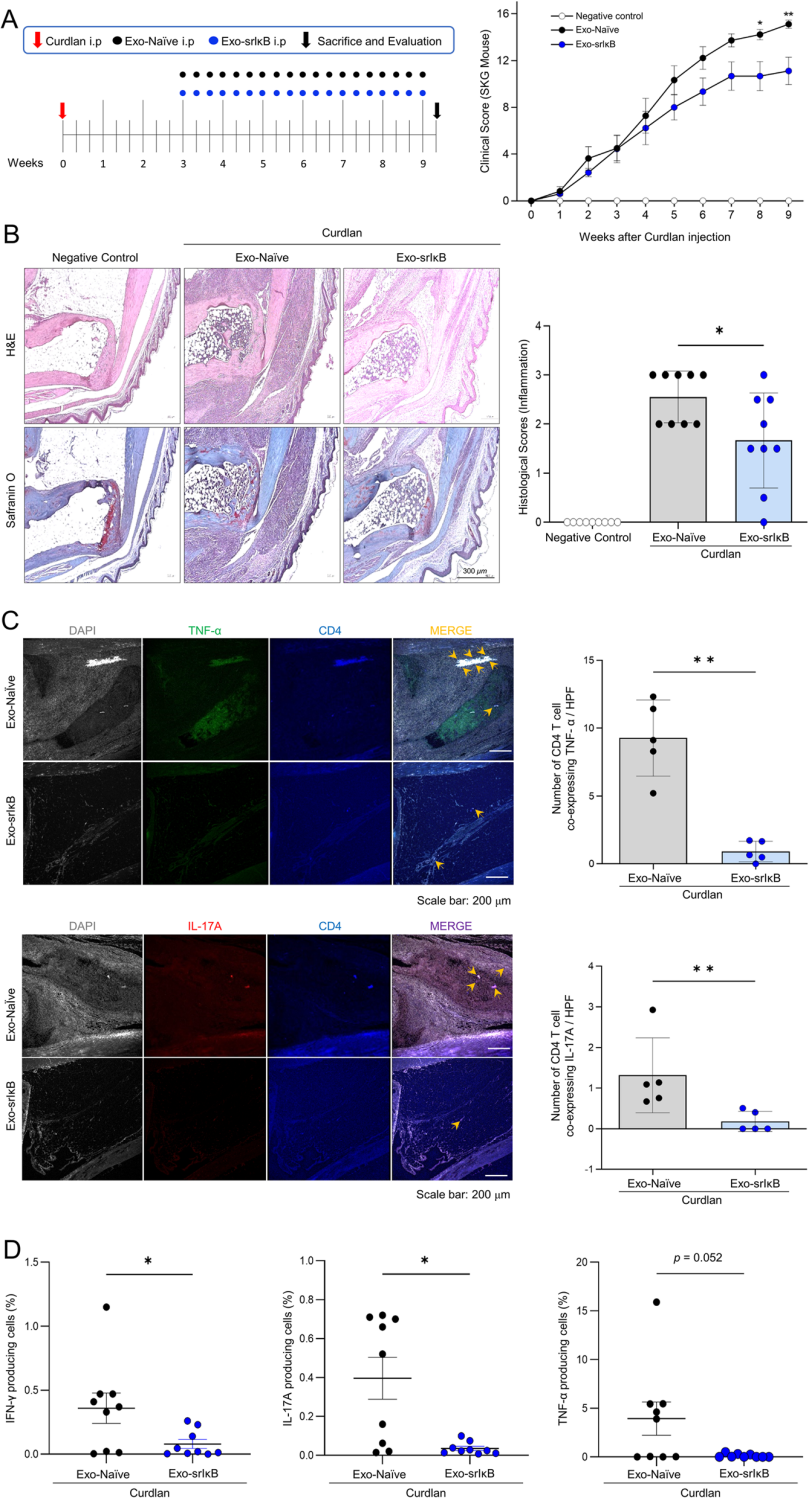
Exo-srIκB demonstrates the ability to suppress arthritis in the *SKG* mouse model. **A** The animal study protocol is depicted, where 11-week-old female SKG mice were treated with either Exo-Naïve or Exo-srIκB starting from the 3rd week after arthritis induction, which was achieved by injecting curdlan. Arthritis scores were assessed based on the clinical severity of arthritis in each group, with a total of nine mice per group. Two-way analysis of variance (ANOVA) was performed to determine statistical significance for the clinical score. **B** A representative tissue stain of the ankle joint at the end of the experiment is displayed in the right panel, along with the analysis of histological scores for inflammation. Kruskal–Wallis test with Dunn’s multiple comparisons was performed to determine statistical significance. **C** The number of cells exhibiting co-expression of TNF-α or IL-17A among CD4-positive T cells per high-power field (HPF) was counted. The prevalence of CD4-positive T cells co-expressing TNF-α or IL-17A was significantly reduced in the ankle joints of mice treated with Exo-srIκB compared to those receiving Exo-Naïve treatment. Over 150 cells in each field were selected, ensuring the exclusion of nonspecific signals. A Mann–Whitney *U*-test was performed to determine statistical significance. **D** Analysis of the frequencies of IFN-γ, IL-17A, and TNF-α-producing cells was conducted on SKG splenocytes. Mann–Whitney *U*-test was performed to determine statistical significance. The values presented are the mean ± SEM, and the symbols represent individual sample. *P < 0.05, **P < 0.01

To explore the impact of Exo-srIκB on arthritis progression in an in vivo model, SKG mice were administered either Exo-Naïve or Exo-srIκB 3 weeks after curdlan injection. Figure 4A (right panel) illustrates the schematic of the study protocol. The Exo-srIκB treatment effectively delayed the onset of arthritis and significantly reduced its severity compared to the Exo-Naïve treatment. Throughout the experiment, Exo-srIκB consistently suppressed arthritis symptoms (Fig. 4A, left panel). Figure 4B (right panel) presents representative histological images of arthritic ankle joints at the end of the experiment. Histological evaluation demonstrated a reduction in inflammatory cell infiltration in mice treated with Exo-srIκB compared to those treated with Exo-Naïve (Fig. 4B, left panel). Under a confocal microscope, immune cells in the ankle joint were observed. The number of CD4-positive T cells co-expressing TNF-α or IL-17A was significantly reduced in the ankle joints of mice treated with Exo-srIκB compared to those receiving Exo-Naïve treatment, as shown in Fig. 4C.

The frequencies of IFN-γ- and IL-17A-producing cells from SKG splenocytes in the Exo-srIκB-treated group showed a significant reduction compared to Exo-Naïve-treated mice. Although the frequency of TNF-α-producing cells did not show a significant difference, there is a noticeable decrease (*p* = 0.052) (Fig. 4D).

### Exo-srIκB treatment suppresses clinical arthritis and reduces the radiographic score of joints in CIA mice

**Fig. 5:**
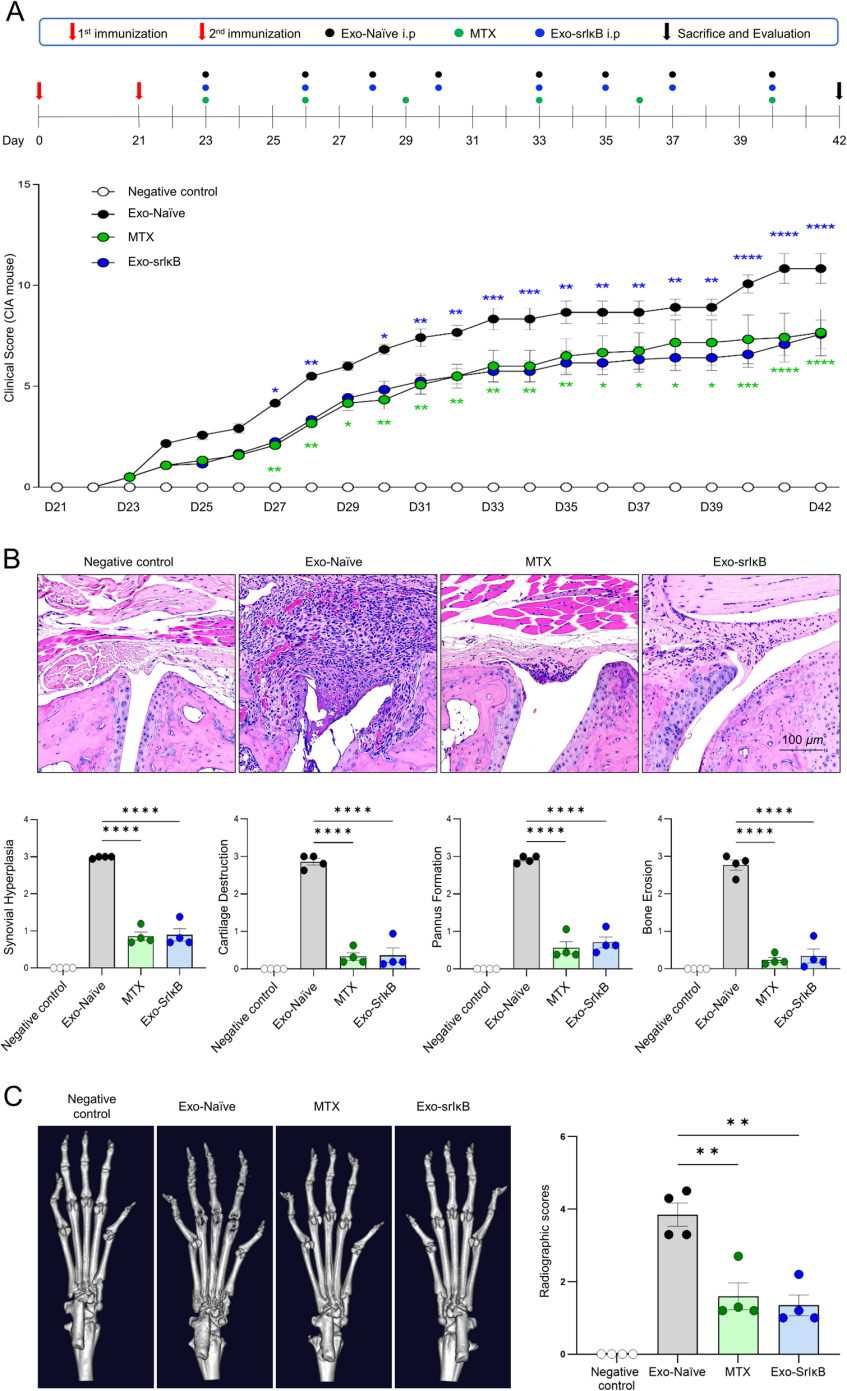
Exo-srIκB exhibits the ability to suppress arthritis and reduce radiographic scores in CIA mouse model. **A** The upper panel shows the study protocol schematic. Arthritis scores were evaluated according to the clinical severity in each group, with five mice per group. Two-way analysis of variance (ANOVA) was performed to determine statistical significance for the clinical score. **B** In the upper panel, a representative tissue stain of the ankle joint at the end of the experiment is displayed. The lower panel presents the analysis of histological scores, including synovial hyperplasia, cartilage destruction, pannus formation, and bone erosion. **C** A representative microCT scan was shown. Radiographic scores were evaluated and analyzed for each group. Mann–Whitney *U*-test or Kruskal–Wallis test with Dunn’s multiple comparisons was performed to determine statistical significance for histologic and radiographic scores. Values are the mean ± SEM. Symbols represent the individual sample. *P < 0.05, **P < 0.01, ***P < 0.001, ****P < 0.0001

The schematic of the study protocol is shown in Fig. 5A (upper panel). The administration of Exo-srIκB demonstrated remarkable efficacy in delaying the onset of arthritis and substantially mitigating its severity when compared to the Exo-Naïve treatment. Notably, Exo-srIκB consistently suppressed arthritis symptoms throughout the experiment, while the MTX treatment comparably suppressed arthritis (Fig. 5A, lower panel). Figure 5B (upper panel) displays representative histological images of arthritic ankle joints at the end of the experiment. Histological evaluation revealed a reduction in synovial hyperplasia, cartilage destruction, pannus formation, and bone erosion in mice treated with Exo-srIκB compared to mice treated with Exo-Naïve (Fig. 5B, lower panel). Radiographic evaluation conducted using microCT demonstrated a reduction in the radiographic score in mice treated with Exo-srIκB, comparable to the MTX-treated group, when compared to mice treated with Exo-Naïve (Fig. 5C).

## Discussion

NF-κB is upregulated in RA synovium and contributes to RA pathology by enhancing the proliferation, invasion, and survival of fibroblast-like synoviocytes [3]. The activation of NF-κB leads to dysregulation of osteoclasts and osteoblasts, resulting in increased bone resorption. Inhibiting NF-κB reduces the secretion of pro-inflammatory mediators and decreases both osteoclast differentiation and bone resorption [16]. Although conventional anti-inflammatory and antirheumatic drugs are known to inhibit NF-κB activation, their potency as NF-κB inhibitors is limited, and they often lack specificity.

Studies using animals with genetically inactivated NF-κB signaling have shown promising results on specific NF-κB inhibition in RA treatment [17]. These genetic engineering studies align well with experiments utilizing highly specific NF-κB inhibitors. Miagkov et al. demonstrated the efficacy of liposomal delivery of NF-κB decoys in preventing the recurrence of streptococcal cell wall arthritis in rats [18]. Similarly, the administration of NF-κB decoys reduced the severity of CIA in rats and suppressed IL-1 and TNF-α production within the joints [19]. Nevertheless, the safety of specific NF-κB inhibitors remains a concern, as NF-κB is crucial for normal development, including the protection of the liver against apoptosis and the immune response against pathogens. Thus, systemic inhibition of the NF-κB pathway could potentially lead to adverse effects [20].

Exosomes are promising for drug delivery due to their safety and unique characteristics. These cell-derived vesicles have natural lipid bilayers, ensuring biocompatibility and minimize immune reactions or toxicity in vivo. Low levels of immunogenic surface proteins allow exosomes to evade immune recognition, thereby reducing associated adverse effects. They possess unique characteristics ideal for protein delivery, including biocompatibility, minimal toxicity, extended circulating half-life, stability, and customizable targeting efficiency [21].

Many types of exosomes derived from body fluids have been found to be immunoregulatory in RA [22, 23]. However, exosomes originating from blood plasma or serum have heterogeneous cellular origins and a poorly defined composition. Utilizing biomimetic exosomes loaded with dexamethasone sodium phosphate nanoparticles effectively enhances the therapeutic impact of glucocorticoids against RA [24]. However, the absence of a separation mechanism between cargo proteins and lipid nanoparticles not only limits the efficiency of cytosolic delivery but also means that the preparation of these particles often involves complicated protein purification steps. Exosomes derived from IL-4 dendritic cells (DCs) exhibit the potential to reduce both the severity and incidence of established CIA [25]. Moreover, administering a single systemic dose of exosomes sourced from IL-10 DCs after the onset of CIA has proven effective in ameliorating disease progression [26]. Research findings indicate that systemic injection of DC/FasL exosomes is an effective treatment for established murine CIA [27]. Furthermore, the injection of miR-150-5p-enriched exosomes derived from mesenchymal stem cells (MSCs) leads to reduce hind paw thickness and improved clinical arthritic scores in a CIA mouse model [28]. However, the use of these genetically modified DC or MSC cells may face stringent regulatory scrutiny; manufacturing standards must all be satisfactorily addressed. In the present study, we used a protein carrier, EXPLOR, which has a higher loading capacity and delivery efficiency [9]. In addition, we examined the efficacy in human samples from patients.

We observed that administering Exo-srIκB did not significantly impact cell viability. Flow cytometry analysis corroborated this, showing no decrease in cell viability for PBMCs and SFMCs. These outcomes suggest that Exo-srIκB does not compromise cell viability. Our findings reveal that Exo-srIκB effectively mitigates inflammatory cytokines in both PBMCs and SFMCs from RA patients. Furthermore, we observed significant improvements in clinical arthritis, inflammatory cytokine production, joint damages, and inflammatory cell infiltration in animal models of RA following the administration of Exo-srIκB. In the subset analysis of PBMCs, Exo-srIκB suppressed inflammation in human monocytes and CD4-positive cells, as indicated by our in vitro results (Suppl. Figure 3A, B). However, it did not produce the same effect on CD8 or mucosal-associated invariant T (MAIT) cells (Suppl. Figure 3C, D).

This study has limitations. We did not thoroughly investigate the effects of Exo-srIκB on specific immune cell subtypes in the ankle joint, apart from CD4 cells. Future investigations should explore the impact of Exo-srIκB on various cell types at inflammatory sites through expanded differentiation and analysis. We need to conduct further research on the specific mechanisms by which Exo-srIκB affects monocytes and CD4-positive T cells in RA. In this pilot study, Exo-srIkB exhibited effectiveness comparable to methotrexate. Additional experiments exploring drug dosage and intervals are necessary to establish a more optimized treatment regimen for RA.

## Conclusion

Our study illustrates the safe and efficient mitigation of arthritis and bone damages in mouse models of RA, achieved by harnessing exosomes as carriers for srIkB. Moreover, Exo-srIkB effectively curtails the levels of inflammatory cytokines in the PBMCs and SFMCs of RA patients. The potential of using exosomes for the direct intracellular delivery of immunosuppressive proteins to target cells presents an innovative avenue for a possible RA treatment approach.

### Supplementary Information

**Additional file 1: Supplementary Fig. 1.** Immunoblot analysis of immune cells. Peripheral blood mononuclear cells were stimulated with PMA and ionomycin and treated with either Exo-Naïve or Exo-srIκB. These cells were then lysed for protein extraction and immunoblotting. Representative results of the immunoblot assay are shown. NF-kB phosphorylation was downregulated. However, other pathways such as p38 and ERK were not affected by Exo-srIκB treatment. **Supplementary Fig. 2.** Schematic plots of the gating strategy for flow cytometry. In flow cytometry analysis, the following gating strategy was employed: first, lymphocytes were gated, then single cells were gated from the lymphocyte population. Subsequently, viable cells were gated, and finally, IL-17A or GM-CSF producing cells were gated. **Supplementary Fig. 3.** Subtype analysis of immune cells. Human monocytic THP-1 cells (5 × 10^5^ cells) were stimulated with LPS (300 ng/mL) and subsequently treated with either PBS, DMSO, an NF-κB inhibitor (as a positive control), or Exo-srIκB. The supernatants were then collected and assayed for TNF-α levels (Suppl. Figure 3A). In a subset analysis of PBMCs, Exo-srIκB was found to suppress inflammation in CD4-positive cells, as indicated by our ex vivo results (Suppl. Figure 3B). However, this effect was not observed in CD8 or MAIT cells (Suppl. Figure 3C, D). Statistical significance was determined using the Mann–Whitney U test or Wilcoxon matched-pairs signed rank test. Each symbol represents an individual sample. NS: not significant; *P < 0.05. Supplementary material. Immunoblot. Sub*set analysis of immune cell by FACS.*

## Data Availability

The data underlying this article will be shared on reasonable request to the corresponding author.

## Abbreviations

RA: Rheumatoid arthritis
NF-κB: Nuclear factor-κB
Exo-srIκB: Exosome-super-repressor lkB
HC: Healthy control
PBMC: Peripheral blood mononuclear cell
SFMC: Synovial fluid mononuclear cell
EVs: Extracellular vesicles
MTX: Methotrexate
MAIT: Mucosal-associated invariant T
Anti-CCP Ab: Anti-cyclic citrullinated peptide antibody
CIBN: N-terminal fragment of cryptochrome-interacting basic-helix-loop-helix 1
