# Supplementary material for: Exosome-mediated delivery of super-repressor IκBα alleviates inflammation and joint damages in rheumatoid arthritis
Source: Arthritis Res Ther. 2024 Jan 2;26:2. doi: 10.1186/s13075-023-03225-1 (PMC10759503; doi:10.1186/s13075-023-03225-1)
Supplement: Supplementary file 1 — Additional file 1: Supplementary Fig. 1. Immunoblot analysis of immune cells. Peripheral blood mononuclear cells were stimulated with PMA and ionomycin and treated with either Exo-Naïve or Exo-srIκB. These cells were then lysed for protein extraction and immunoblotting. Representative results of the immunoblot assay are shown. NF-kB phosphorylation was downregulated. However, other pathways such as p38 and ERK were not affected by Exo-srIκB treatment. Supplementary Fig. 2. Schematic plots of the gating strategy for flow cytometry. In flow cytometry analysis, the following gating strategy was employed: first, lymphocytes were gated, then single cells were gated from the lymphocyte population. Subsequently, viable cells were gated, and finally, IL-17A or GM-CSF producing cells were gated. Supplementary Fig. 3. Subtype analysis of immune cells. Human monocytic THP-1 cells (5 × 10^5^ cells) were stimulated with LPS (300 ng/mL) and subsequently treated with either PBS, DMSO, an NF-κB inhibitor (as a positive control), or Exo-srIκB. The supernatants were then collected and assayed for TNF-α levels (Suppl. Figure 3A). In a subset analysis of PBMCs, Exo-srIκB was found to suppress inflammation in CD4-positive cells, as indicated by our ex vivo results (Suppl. Figure 3B). However, this effect was not observed in CD8 or MAIT cells (Suppl. Figure 3C, D). Statistical significance was determined using the Mann–Whitney U test or Wilcoxon matched-pairs signed rank test. Each symbol represents an individual sample. NS: not significant; *P < 0.05. Supplementary material. Immunoblot. Subset analysis of immune cell by FACS. [file 13075_2023_3225_MOESM1_ESM.zip › Supplementary material and methods.docx]

**Supplementary figure legends**

**Supplementary Figure 1. Immunoblot analysis of immune cells**

Peripheral blood mononuclear cells were stimulated with PMA and ionomycin and treated with either Exo-Naïve or Exo-srIκB. These cells were then lysed for protein extraction and immunoblotting. Representative results of the immunoblot assay are shown. NF-kB phosphorylation was downregulated. However, other pathways such as p38 and ERK were not affected by Exo-srIκB treatment

**Supplementary Figure 2. Schematic plots of the gating strategy for flow cytometry**

In flow cytometry analysis, the following gating strategy was employed: first, lymphocytes were gated, then single cells were gated from the lymphocyte population. Subsequently, viable cells were gated, and finally, IL-17A or GM-CSF producing cells were gated.

**Supplementary Figure 3. Subtype analysis of immune cells**

Human monocytic THP-1 cells (5 x 10^5^ cells) were stimulated with LPS (300 ng/mL) and subsequently treated with either PBS, DMSO, an NF-κB inhibitor (as a positive control), or Exo-srIκB. The supernatants were then collected and assayed for TNF-α levels (Suppl. Figure 3A). In a subset analysis of PBMCs, Exo-srIκB was found to suppress inflammation in CD4-positive cells, as indicated by our *ex vivo* results (Suppl. Figure 3B). However, this effect was not observed in CD8 or MAIT cells (Suppl. Figure 3C, D). Statistical significance was determined using the Mann-Whitney U test or Wilcoxon matched-pairs signed rank test. Each symbol represents an individual sample. NS: not significant; *P < 0.05.

Supplementary Material

**Immunoblot**

Peripheral blood mononuclear cells were isolated and cultured in RPMI1640 media (LM011-01, welgene, Korea) supplemented with 10% Fetal Bovine Serum (S001-01, welgene, Korea) and 1% penicillin-streptomycin solutions (LS202-02, welgene, Korea). Cells were seeded at a density of 5 x 105 cells/well in a 96-well plate. After a 3-hour pretreatment with Exo-Naïve or Exo-srIκB, cells were stimulated with Phorbol 12-Myristate 13-acetate (PMA; P1585, Sigma, USA) at a concentration of 100 ng/ml, Ionomycin (I9657, Sigma, USA) at a concentration of 1 μM. Cells were lysed with 1X RIPA buffer containing phosphatase (5870S, Cells signaling, USA) and protease (535140, Calbiochem, USA) inhibitors. Proteins were quantified with a Bradford assay. Protein (10-30 μg) were subjected to immunoblotting. Antibodies used for immunoblotting were as follows: phos-NF-kB p65 (3033), phos-p38 (9295), phos-ERK (9101), total-ERK (9102), and GAPDH (2118) from Cell Signaling Technology (Danvers, MA, USA). Total NF-kB p65 (sc-372) and Total-p38 (sc-535) antibody was purchased from Santa Cruz Biotechnology (Dallas, TX, USA). Secondary antibody for goat anti-rabbit IgG (111-035-003) and goat anti-mouse IgG (115-035-003) were obtained from Jackson ImmunoResearch (West Grove, PA, USA)

**Subset analysis of immune cell by FACS**

Peripheral blood mononuclear cells were seeded at a density of 5 x 10^5^ cells/well in a 96-well plate. After a 3-hour pretreatment with Exo-srIκB, cells were stimulated with Phorbol 12-Myristate 13-acetate (PMA; P1585, Sigma, USA) at a concentration of 100 ng/ml, Ionomycin (I9657, Sigma, USA) at a concentration of 1 μM, and Brefeldin A (Golgiplug-protein transport inhibitor; 555029, BD, USA). The cells were then incubated in CO_2_, 37 °C incubator for 4 hours. Following stimulation, cells were stained with anti-Fixable Viability Dye-eFuor780 (65-0865-14, Invitrogen, USA) and pacific Blue anti- human CD4 (300520, BioLegend, USA), APC Mouse Anti-Human CD8 (561952, BD, USA), APC-Cy™7 Mouse Anti-Human CD3 (341090, BD, USA), PE-cy 5 Mouse Anti human CD161 (551138, BD pharmingen, USA) and APC anti human TCR Va7.2 (351708, BioLegend, USA), After washing, cells were fixed and permeabilized using Perm/wash buffer and stained with PerCP-Cy5.5-conjugated anti-GM-CSF (502312, BioLegend, USA), PE-Cy™7 Mouse Anti-Human TNF (557647, BD biosciences, USA) antibodies.
