# Supplementary figures and images for: Exosome-mediated delivery of super-repressor IκBα alleviates inflammation and joint damages in rheumatoid arthritis
Source: Arthritis Res Ther. 2024 Jan 2;26:2. doi: 10.1186/s13075-023-03225-1 (PMC10759503; doi:10.1186/s13075-023-03225-1)

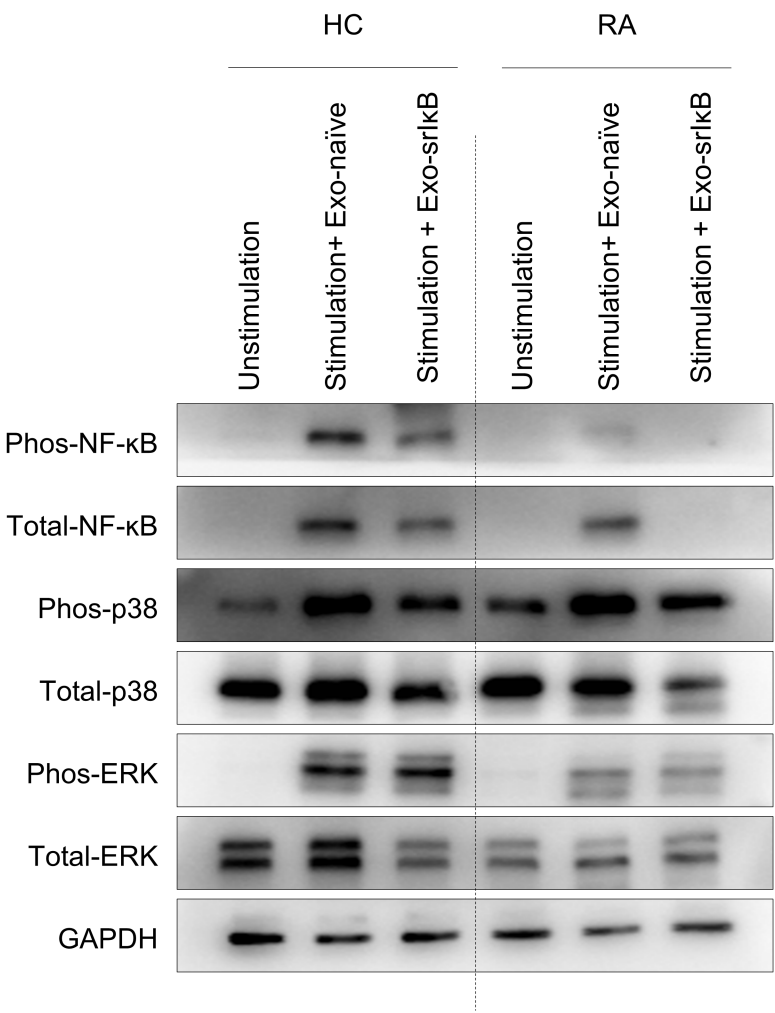

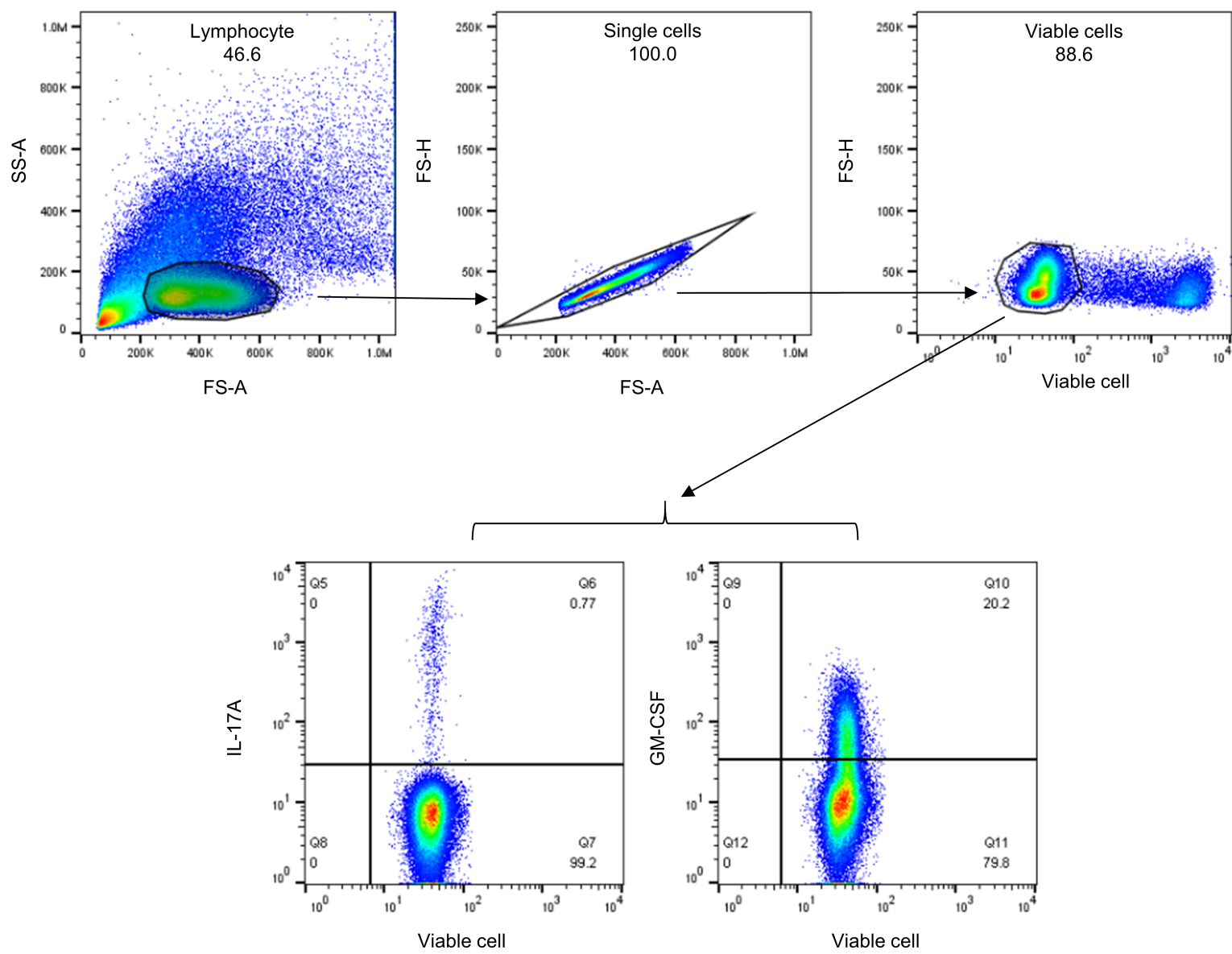

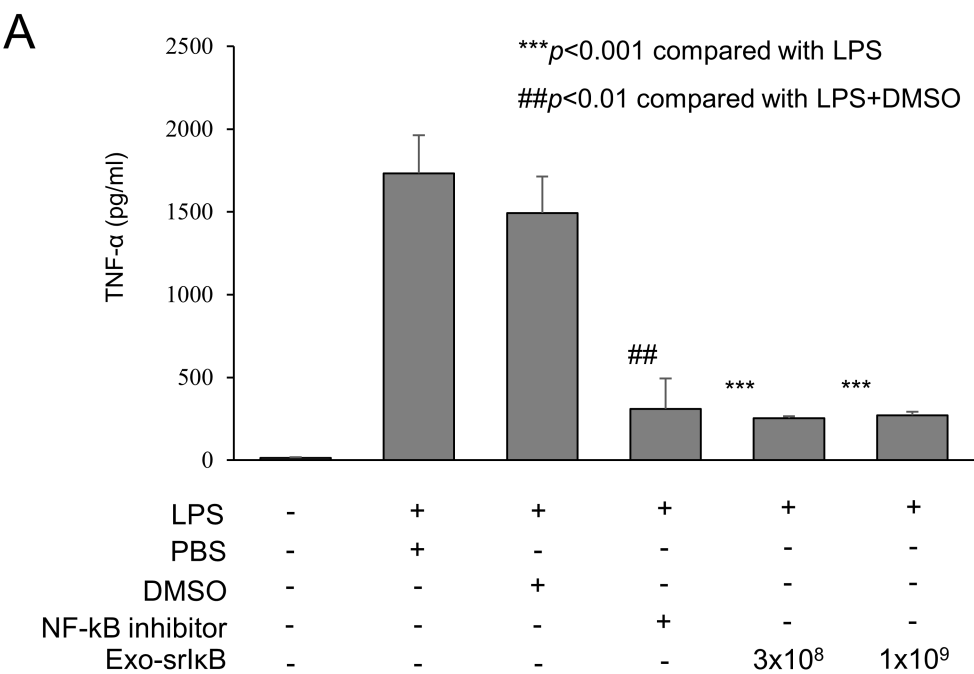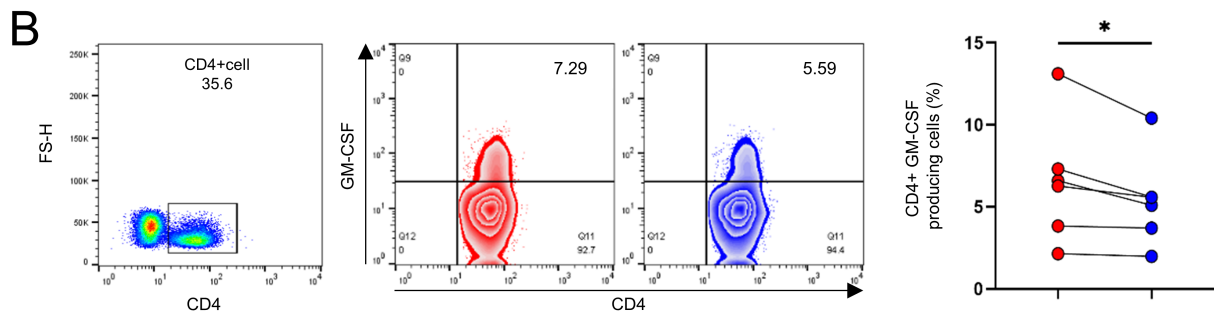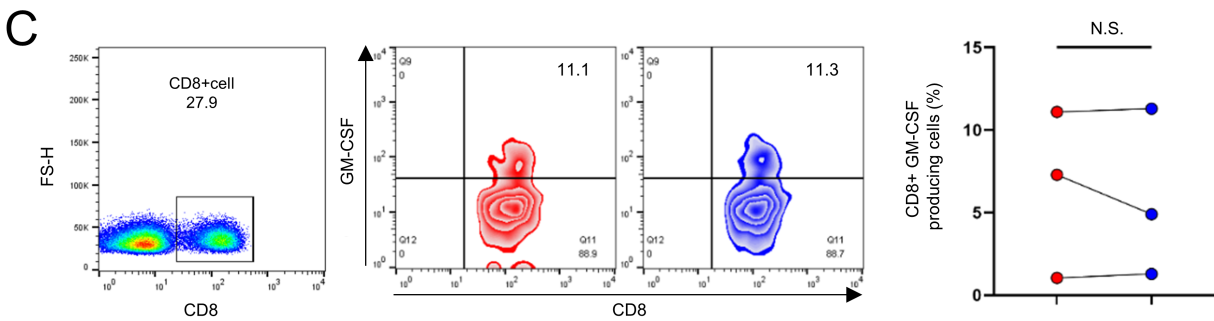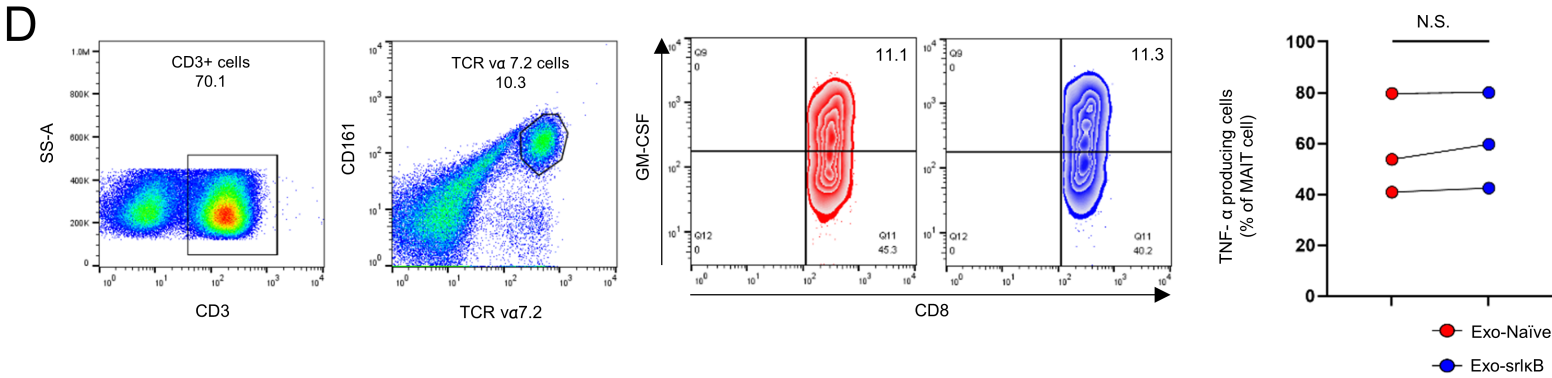

Supplement: Supplementary file 1 — Additional file 1: Supplementary Fig. 1. Immunoblot analysis of immune cells. Peripheral blood mononuclear cells were stimulated with PMA and ionomycin and treated with either Exo-Naïve or Exo-srIκB. These cells were then lysed for protein extraction and immunoblotting. Representative results of the immunoblot assay are shown. NF-kB phosphorylation was downregulated. However, other pathways such as p38 and ERK were not affected by Exo-srIκB treatment. Supplementary Fig. 2. Schematic plots of the gating strategy for flow cytometry. In flow cytometry analysis, the following gating strategy was employed: first, lymphocytes were gated, then single cells were gated from the lymphocyte population. Subsequently, viable cells were gated, and finally, IL-17A or GM-CSF producing cells were gated. Supplementary Fig. 3. Subtype analysis of immune cells. Human monocytic THP-1 cells (5 × 10^5^ cells) were stimulated with LPS (300 ng/mL) and subsequently treated with either PBS, DMSO, an NF-κB inhibitor (as a positive control), or Exo-srIκB. The supernatants were then collected and assayed for TNF-α levels (Suppl. Figure 3A). In a subset analysis of PBMCs, Exo-srIκB was found to suppress inflammation in CD4-positive cells, as indicated by our ex vivo results (Suppl. Figure 3B). However, this effect was not observed in CD8 or MAIT cells (Suppl. Figure 3C, D). Statistical significance was determined using the Mann–Whitney U test or Wilcoxon matched-pairs signed rank test. Each symbol represents an individual sample. NS: not significant; *P < 0.05. Supplementary material. Immunoblot. Subset analysis of immune cell by FACS. [file 13075_2023_3225_MOESM1_ESM.zip › suppl.figure.pdf]
